# The Dispensatory of the United States of America

**Published:** 1883-06

**Authors:** 


**The Dispensatory of the United States of America.** By Dr. GEORGE B. WOOD and Dr. FRANKLIN BACHE. Fifteenth edition; rearranged, thoroughly revised and largely rewritten, with illustrations. By H. C. WOOD, M. D., Professor of Materia Medica and Therapeutics, and of Diseases of the Nervous System, in the University of Pennsylvania; JOSEPH P. REMTON, Ph. D., Professor of Theory and Practice of Pharmacy in the Philadelphia College of Pharmacy: and SAMUEL P. SADTLER, Ph. D., F. C. S., Professor of Chemistry in the Philadelphia College of Pharmacy. Philadelphia: J. P. Lippincott & Co. 1883. Price $8.00.

It is just fifty years ago that Dr. George B. Wood gave to the American profession the first edition of the U. S. Dispensatory, and from that day to this the respective editions have been universally accepted as the standard commentary on the official Pharmacopœia, and it has been so employed, not only in this country but in every land where the English language is spoken. Such a success is, we believe, unrivaled in medical literature, and is to be accounted for only by the thoroughness, accuracy and completeness which has characterized the work, and to the energy and zeal of its editors, who have never allowed the work to fall behind the progress of the age. The present edition is the fifteenth, and the highest praise we can give it is to say that in value it surpasses its predecessors. The advance in the last decade in medical, pharmaceutical and chemical knowledge has necessitated many changes and additions, and these have been made with so free a hand that the present volume may be considered a new book, founded upon the old U. S. Dispensatory. We have read but a few of its 2,000 pages, but those articles which we have had time to peruse have shown us that the very latest achievements in pharmacology are recorded in this volume. A novel feature in this edition is the indication of the pronunciation of the officinal titles by diacritical marks. This addition will, we hope, lessen the cacœpy so painfully common, not only among the druggists but also with medical men, who have less excuse for mispronunciation. There has been added, also, a very complete list of analyses of American mineral spring waters, and some of European note. The illustrations are new and original; and in every way the book is worthy of the universal commendation with which this edition has been received.

